# Complete chloroplast genome sequence of *Stachys japonica* (Labiatae)

**DOI:** 10.1080/23802359.2020.1787263

**Published:** 2020-07-07

**Authors:** Mingcheng Wang, Qi Zhao, Dechun Jiang, Zhiqiang Wang

**Affiliations:** a Institute for Advanced Study, Chengdu University, Chengdu, China; b CAS Key laboratory of Mountain Ecological Restoration and Bioresource Utilization & Ecological Restoration and Biodiversity Conservation Key Laboratory of Sichuan Province, Chengdu Institute of Biology, Chinese Academy of Sciences, Chengdu, China

**Keywords:** *Stachys japonica;* chloroplast genome, phylogenetic analyses

## Abstract

The complete chloroplast genome of *Stachys japonica* was reconstructed by reference-based assembly using Illumina paired-end data. The assembled plastome is 150,599 base pairs (bp) in length, including a pair of inverted repeat regions (IRs) of 25,654 bp each, a large single-copy region (LSC) of 81,701 bp and a small single-copy region (SSC) of 17,590 bp. A total of 131 genes were predicted from the chloroplast genome, including 86 protein coding genes, 37 tRNA genes and 8 rRNA genes. The overall GC content of *S. japonica* chloroplast genome was 38.5%. Phylogenetic analysis with several reported chloroplast genomes showed that *S. japonica* is closely clustered with *S. sylvatica*. The complete chloroplast genome of *S. japonica* provides new insight into Labiatae evolutionary and genomic studies.

The perennial herb *Stachys japonica* is widely distributed in China, Japan and Russia. It has been used in traditional Chinese medicine for the treatment of dysentery, laryngitis and a few other diseases. To date, the phylogenetic position of *S. japonica* in the genus *Stachys* is still unclear. In this study, we first reported the complete chloroplast (cp) genome of *S. japonica* and reconstructed a plastome phylogeny for the genus *Stachys*.

The mature and healthy leaves of a single individual of *S. japonica* was sampled from the field of Haiyuan county in south Ningxia, NW China (36°12′43″N, 105°37′9″E). The voucher specimen was deposited in the Herbarium of Sichuan University (accession number: QTPLJQ14383116). The total genomic DNA was extracted from silica gel dried leaves using a modified CTAB method (Doyle and Doyle 1987) and sequenced based on the Illumina pair-end technology. The filtered reads were assembled using the program NOVOPlasty (Dierckxsens et al. 2017) with complete cp genome of *S. coccinea* as the reference (GenBank accession no. NC_029823). The assembled cp genome was annotated using Plann (Huang and Cronk 2015), and the annotation was corrected using Geneious (Kearse et al. 2012). To examine the phylogenetic position of *S. japonica*, a multiple sequence alignment (MSA) analyses was performed using MAFFT v7.313 (Katoh and Standley 2013) based on seven cp genomes in Labiatae. Finally, a maximum likelihood (ML) tree was constructed by RAxML v8.2.11 (Stamatakis 2006) with 500 bootstrap replicates based on the alignments, using two species from the genus *Lamium* as outgroup.

The complete cp genome of *S. japonica* was a circular molecular genome with a size of 150,599 bp in length, which presented a typical quadripartite structure containing two inverted repeat (IR) regions of 25,654 bp separated by the large single-copy (LSC) region of 81,701 bp and small single-copy (SSC) region of 17,590 bp. The cp genome consists of 131 genes including 86 protein coding genes, 37 tRNA genes, and 8 rRNA genes. The overall GC content was about 38.5%. In the plastome phylogeny, *S. japonica* shows the closest genetic relationship to *S. sylvatica*. (100% bootstrap support) (Figure 1). The *S. japonica* cp genome can be further used for population genomic studies, phylogenetic analyses, genetic engineering studies of Labiatae.

**Figure 1.**
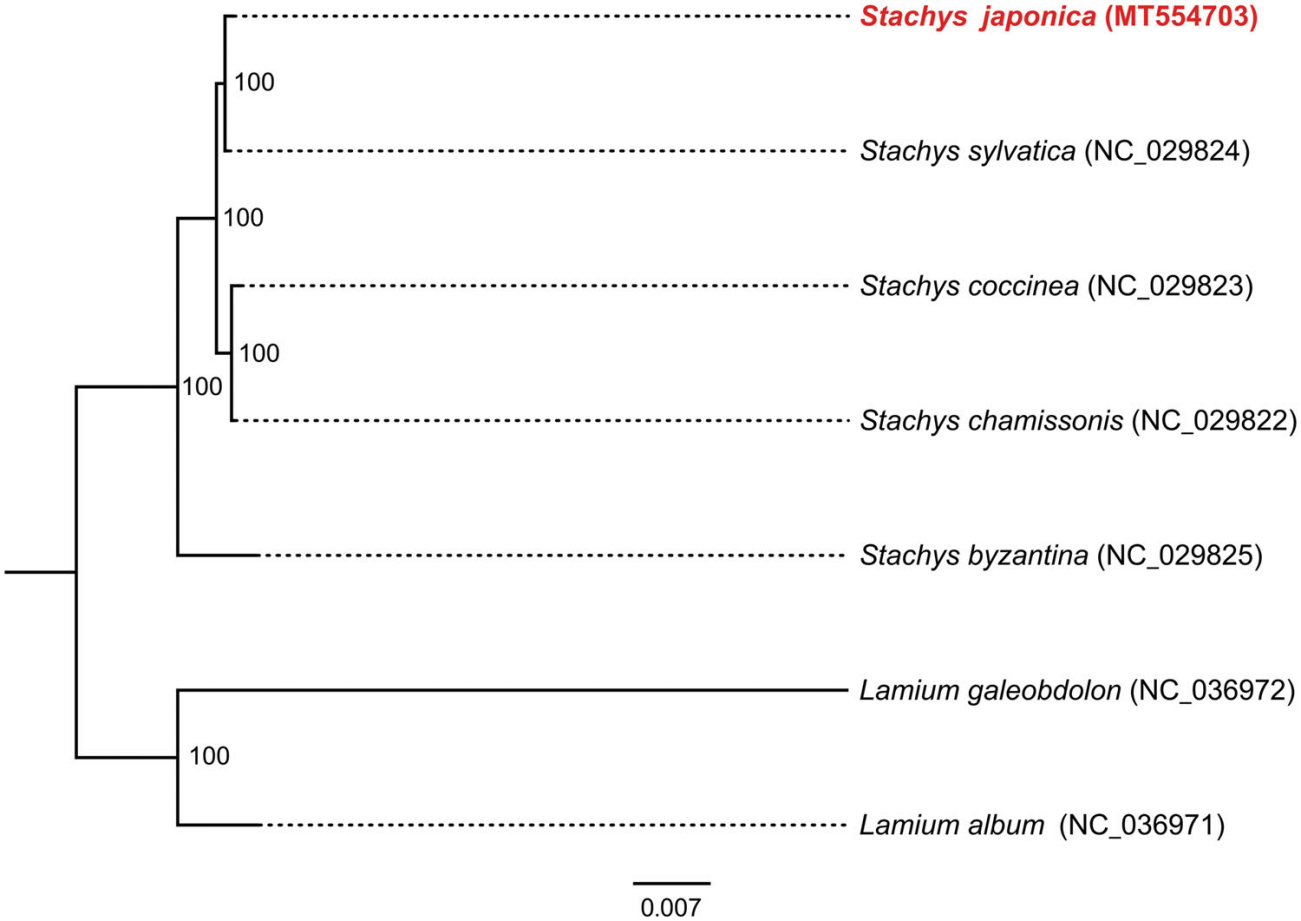
Phylogenetic relationships of seven species based on chloroplast genome sequences. Bootstrap support is indicated for each branch..

## Data Availability

The data that support the findings of this study are openly available in NCBI GenBank database at https://www.ncbi.nlm.nih.gov/ with the accession number is MT554703 or available in [figshare.com] at https://doi.org/10.6084/m9.figshare.12443378.
